# Supportive oncology care at home interventions: protocols for clinical trials to shift the paradigm of care for patients with cancer

**DOI:** 10.1186/s12885-022-09461-z

**Published:** 2022-04-09

**Authors:** Ryan D. Nipp, Eliza Shulman, Melissa Smith, Patricia M. C. Brown, P. Connor Johnson, Eva Gaufberg, Charu Vyas, Carolyn L. Qian, Isabel Neckermann, Shira B. Hornstein, Mathew J. Reynolds, Joseph Greer, Jennifer S. Temel, Areej El-Jawahri

**Affiliations:** 1 Massachusetts General Hospital, 55 Fruit Street, Yawkey 9E, Boston, MA 02114 USA; 2 Harvard Medical School, Boston, MA USA; 3 Medically Home, Boston, MA USA

**Keywords:** Hospital at home care, Oncology care at home, Supportive oncology care at home

## Abstract

**Background:**

Patients with cancer often endure substantial symptoms and treatment toxicities leading to high healthcare utilization, including hospitalizations and emergency department visits, throughout the continuum of their illness. Innovative oncology care models are needed to improve patient outcomes and reduce their healthcare utilization. Using a novel hospital at home care platform, we developed a Supportive Oncology Care at Home intervention to address the needs of patients with cancer.

**Methods:**

We are conducting three trials to delineate the role of Supportive Oncology Care at Home for patients with cancer. The Supportive Oncology Care at Home intervention includes: (1) a hospital at home care model for symptom assessment and management; (2) remote monitoring of daily patient-reported symptoms, vital signs, and body weight; and (3) structured communication with the oncology team. Our first study is a randomized controlled trial to test the efficacy of Supportive Oncology Care at Home versus standard oncology care for improving healthcare utilization, cancer treatment interruptions, and patient-reported outcomes in patients with cancer receiving definitive treatment of their cancer. Participants include adult patients with gastrointestinal and head and neck cancer, as well as lymphoma, receiving definitive treatment (e.g., treatment with curative intent). The second study is a single-arm trial assessing the feasibility and acceptability of the Supportive Oncology Care at Home intervention for hospitalized patients with advanced cancer. Eligible participants include adult patients with incurable cancer who are admitted with an unplanned hospitalization. The third study is a single-arm trial assessing the feasibility and acceptability of the Supportive Oncology Care at Home intervention to enhance the end-of-life care for patients with advanced hematologic malignancies. Eligible participants include adult patients with relapsed or refractory hematologic malignancy receiving palliative therapy or supportive care alone.

**Discussion:**

These studies are approved by the Dana-Farber/Harvard Cancer Center Institutional Review Board and are being conducted in accordance with the Consolidated Standards of Reporting Trials statement for non-pharmacological trials. This work has the potential to transform the paradigm of care for patients with cancer by providing them with the necessary support at home to improve their health outcomes and care delivery.

**Trial registrations:**

NCT04544046, NCT04637035, NCT04690205.

## Background

Patients with cancer endure a serious, life-threatening diagnosis and often receive intensive therapies that result in substantial side-effects and toxicities such as nausea, vomiting, diarrhea, fatigue, and infectious complications [1–7]. These toxicities commonly lead to high healthcare utilization, including frequent emergency department (ED) visits and prolonged hospitalizations, which contribute to the rising costs of cancer care, impaired patient quality of life (QOL), and morbidity related to treatment [1–16]. Notably, patients with advanced cancer experience multiple ED visits during the first year of diagnosis, with over half of these visits resulting in an inpatient hospitalization [4–6, 17]. Moreover, over 40% of hospitalized patients with advanced cancer have a hospital readmission within 90 days of discharge and a substantial proportion of these hospitalizations are potentially avoidable [13–15]. Thus, there is a critical need to develop novel healthcare delivery models to enhance the experience of patients with cancer, improve patient outcomes, and reduce their healthcare utilization.

The hospital at home care model offers an alternative approach to treating patients in need of emergent or inpatient acute care in their homes, with many studies demonstrating its safety and efficacy, albeit in the general medical population [18–30]. Hospital at home entails the provision of comprehensive medical care, such as vital sign monitoring, clinician home visits, intravenous therapies, physical therapy, and nutritional services, coupled with a rapid response to address the needs of acutely ill patients in their home [21, 28–30]. Prior research demonstrates that hospital at home care models improve patient outcomes, such as satisfaction with care and functional status, while decreasing healthcare utilization and costs in patients with chronic medical conditions, such as congestive heart failure and chronic obstructive lung disease [18–30]. A recent study of a hospital at home care model replacing acute hospitalizations for patients with cancer has also shown a reduction in healthcare utilization and cost of care [31]. However, hospital at home interventions have not been tested as longitudinal care models for patients with cancer to improve their overall QOL and care throughout their illness continuum. Thus, hospital at home longitudinal care models represent an innovative approach with the potential to improve clinical outcomes, optimize cancer care delivery, and reduce healthcare utilization among patients with cancer.

Symptom monitoring interventions in oncology have demonstrated encouraging efficacy in reducing patients’ symptom burden, improving their QOL, and decreasing healthcare utilization [32–35]. For example, randomized trials of symptom monitoring interventions in ambulatory patients with cancer have shown better symptom control, decreased use of hospital-level care, and improved survival for those receiving the intervention compared to usual care [32–37]. Therefore, integrating symptom monitoring with hospital at home care models provides a novel solution to help optimize the care of patients with cancer, enhance their clinical outcomes, and reduce their use of healthcare services.

Patients with cancer experience immense toxicities and a substantial hospitalization burden, and we therefore developed a novel Supportive Oncology Care at Home program to address patients’ symptoms and clinical needs, improve their QOL and care experience, and reduce their use of hospital-level care. The Supportive Oncology Care at Home intervention includes the following: (1) hospital at home care model for symptom assessment and management; (2) remote monitoring of patient-reported symptoms, vital signs, and body weight; and (3) structured communication with the oncology team. We are currently conducting three trials to delineate the role of Supportive Oncology Care at Home in addressing the needs of patients with cancer throughout the continuum of their illness.

## Methods/design

### The supportive oncology care at home intervention

**Fig. 1:**
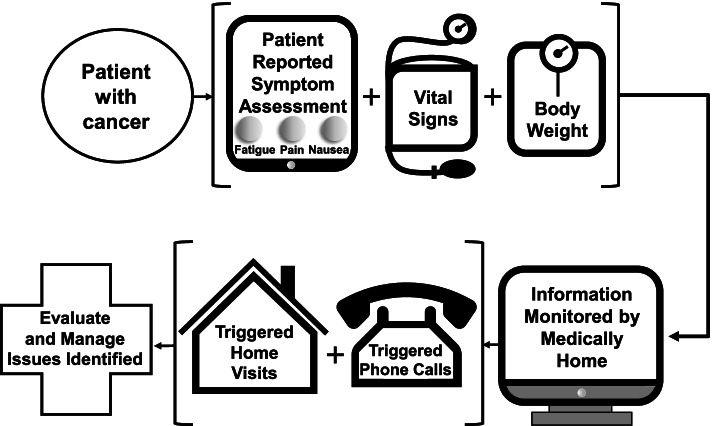
Supportive Oncology Care at Home Intervention

We developed the Supportive Oncology Care at Home intervention with input from oncology nurses, nurse practitioners, oncologists, and palliative care clinicians who specialize in caring for patients with cancer (Fig. 1). The Supportive Oncology Care at Home intervention is provided by a dedicated, trained Medically Home care team (physicians, advance practice clinicians, and nurses) in collaboration and partnership with the primary oncology team. Medically Home is available 24-h a day to provide home assessments and deliver needed interventions at home, including radiology studies, laboratory tests, intravenous hydration, medications, and rapid response, as needed. Additional services including physical and occupational therapy, nutrition, home health aides, durable medical equipment, and social work are also available to help meet the needs of patients and their families within their home. Patients receive a Medically Home technology platform that supports virtual care, including video visits, patient data transmission, and deployment of home-based services.

Patients receiving the intervention first have an initial one-hour visit in their home with the Medically Home team. During this visit, a trained Medically Home clinician reviews the services included in the program, performs a physical exam, assesses the patient’s home safety, and educates the patient and caregivers on the use of technology provided (e.g., tablet computer, wireless phone, and vital sign monitoring equipment). The Medically Home care team provides: (1) monitoring of patient-reported symptoms using the Edmonton Symptom Assessment System (ESAS), vital signs, and body weight measurement with appropriate triggers for phone calls and home visits by Medically Home based on a clinician-derived algorithm; (2) clinician home visits for medical assessment and management as needed; and (3) regular communication with oncology clinicians regarding care delivered at home to ensure continuity of care. Patient reports of their symptoms and vital signs are monitored in real-time and reviewed by Medically Home clinicians to initiate prompt responses. Using data from the symptom, vital sign, and body weight monitoring, the intervention contains detailed algorithms indicating when the Medically Home team should call the patient to check-in. For example, Medically Home can contact the patient regarding any of the following: (1) if the patient does not complete their daily symptom assessment by 1:00 pm; (2) any ESAS symptom score ≥ 7; (3) an increase in ESAS symptoms score ≥ 2 points from the previous day; (4) heart rate < 50 or > 100 beats per minute; if baseline heart rate > 100, then a 25-point increase in heart rate should result in a phone call; (5) temperature > 100.4 degrees Fahrenheit; (6) oxygen saturation < 90%; and (7) weight loss of 5 or more pounds over the prior week. If the patient has questions or concerns at any time, they can contact Medically Home directly via a 24/7 access line. The study team and Medically Home clinicians meet weekly to help ensure intervention fidelity, adherence, and utility.

### Study #1: supportive oncology care at home for patients with cancer receiving definitive treatment

#### Study design/objectives

**Fig. 2:**
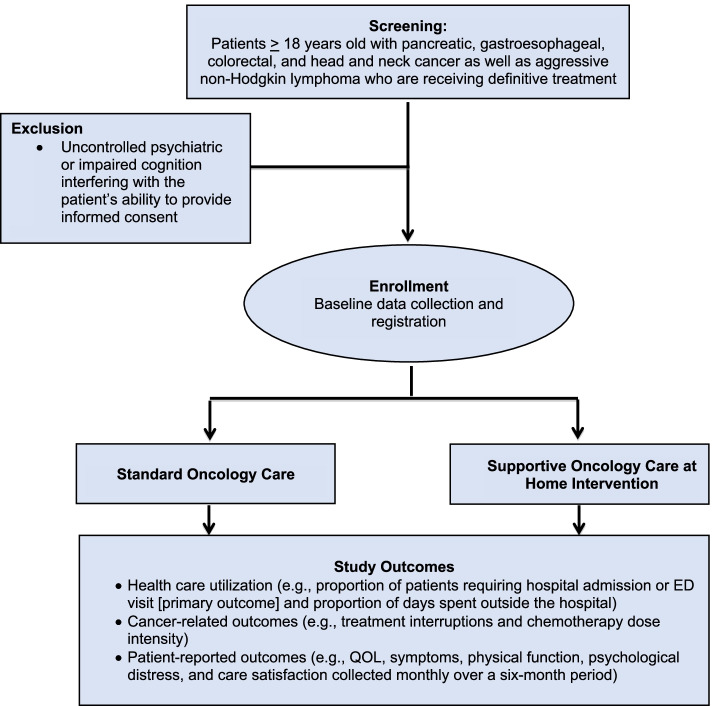
Supportive Oncology Care at Home for Patients with Cancer Receiving Definitive Treatment Consort Diagram

We recently completed a pilot feasibility study assessing the role of Supportive Oncology Care at Home for patients with pancreatic cancer receiving neoadjuvant treatment [38]. Findings of this pilot demonstrated the feasibility and acceptability of the Supportive Oncology Care at Home intervention, with encouraging preliminary findings demonstrating reduced healthcare utilization in this population. Building on this work, we are currently conducting a randomized controlled trial to assess the efficacy of the Supportive Oncology Care at Home intervention for enhancing care delivery and outcomes in 300 patients with cancer receiving definitive treatment with curative intent (Fig. 2) (NCT04544046; DFCI Protocol Number:20–331). We are recruiting patients at Massachusetts General Hospital (MGH) in Boston and two MGH community affiliates (MGH Newton-Wellesley and MGH North Shore). This study is approved by the Dana-Farber/Harvard Cancer Center (DF/HCC) Institutional Review Board. Study staff received detailed training in data entry, security, storage, and management procedures for data collection.

#### Participant selection

The study focuses on oncology populations receiving definitive treatment who have high-risk for side effects, treatment interruptions, ED visits, and hospitalizations during treatment. To be eligible, patients must be: (1) age 18 or older; (2) receiving definitive treatment (i.e., chemotherapy and/or chemoradiation with curative intent) for pancreatic cancer, gastroesophageal cancer, colorectal cancer, head and neck cancer, or aggressive non-Hodgkin lymphoma; (3) within two weeks of starting treatment; (4) planning to receive care at MGH or MGH affiliate sites; (5) verbally fluent in English; and (6) residing in-state, within approximately 50 miles of MGH. Exclusion criteria include uncontrolled psychiatric or impaired cognition interfering with the patient’s ability to understand study procedures and provide informed consent based on the oncology clinician’s assessment.

#### Enrollment and randomization

We will identify patients for study participation by screening the outpatient gastrointestinal oncology, head and neck cancer, and lymphoma clinic schedules and clinical trial enrollment on a weekly basis. The study team will email the oncology clinician to request permission to approach the patient for study participation. If the oncology clinician has no objections, the study team will approach the patient in-person or over the phone and obtain informed consent. Patients will then be asked to complete baseline assessment and they will be registered and randomized using a central randomization office. Randomization will be 1:1, stratified by cancer type (gastroesophageal vs. pancreatic vs. rectal vs. head and neck vs. lymphoma) to the Supportive Oncology Care at Home intervention described above (Fig. 1) versus usual care (computer generated with randomly permuted blocks). Participants assigned to usual care will receive standard oncology care and attend their regular clinic visits.

#### Timeline

The duration of an individual’s active participation in the study will include their time receiving definitive treatment (i.e., neoadjuvant chemotherapy or chemoradiation treatment with curative intent) and up to 6 months following the treatment start date. For those with gastrointestinal cancer, the Supportive Oncology Care at Home intervention will continue throughout the duration of their chemotherapy treatment. For those with head and neck cancer, the intervention will continue throughout the duration of their chemoradiation treatment and until 30 days after completing treatment, given the residual symptoms experienced by this population.

#### Study outcomes

The primary endpoint of the trial is the proportion of patients requiring hospital admission or ED visit (yes vs. no) during the study period. Secondary endpoints include: (1) proportion of days patients spent outside of the hospital during the study period; (2) proportion of patients needing an urgent visit to the clinic (yes vs. no); (3) proportion of patients requiring treatment interruption (yes vs. no); (4) relative dose intensity of definitive treatment received; and (5) change in patient-reported outcomes longitudinally during the study period (up to six months after therapy initiation). Patient-reported outcomes will be collected at baseline and monthly throughout the intervention until six months post-baseline. Table 1 depicts study outcomes including the patient-reported assessments used in this trial.

**Table 1:** Supportive Oncology Care at Home for Patients with Cancer Receiving Definitive Treatment Study Outcomes

Study Outcomes
**Health Care Utilization Outcomes**
Proportion of patients requiring a hospital admission or ED visit during the study period **(primary endpoint)**
Proportion of days patients spent outside of the hospital during the study period
Patients needing an urgent care visit (yes/no)
**Cancer treatment-related outcomes**
Patients receiving treatment interruption (yes/no)
Relative dose intensity of definitive treatment received
**Patient-reported outcomes**
Changes in symptom burden (ESAS) longitudinally throughout the study
Changes in QOL (FACT-G) longitudinally throughout the study
Changes in psychological distress (HADS/PHQ-4) longitudinally throughout the study
Changes in care satisfaction (FAMCARE) longitudinally throughout the study
Changes in ADLs (MOS) and IADLs (OARS) longitudinally throughout the study

#### Sample size calculation

The primary endpoint of the proposed study is a comparison of the proportion of patients requiring a hospital admission or ED visit during the study period between the study groups. Enrolling 300 patients, or 150 per arm, will provide > 80% power to detect a 15% difference between arms, assuming the rate in the control arm is 55% (based on our experience in the pilot study and prior published literature), with a 5% two-sided type 1 error. A 15% difference in the proportion of patients requiring a hospital admission or ED visit represents a clinically important difference, consistent with other practice-changing supportive care interventions in oncology [32, 39–41]. We had no missing data on healthcare utilization in our pilot work as these patients are receiving care at our institution, and we will use the intention-to-treat principle with all randomized subjects.

### Study #2: feasibility of delivering a supportive oncology care at home intervention for hospitalized patients with cancer

#### Study design/objectives

**Fig. 3:**
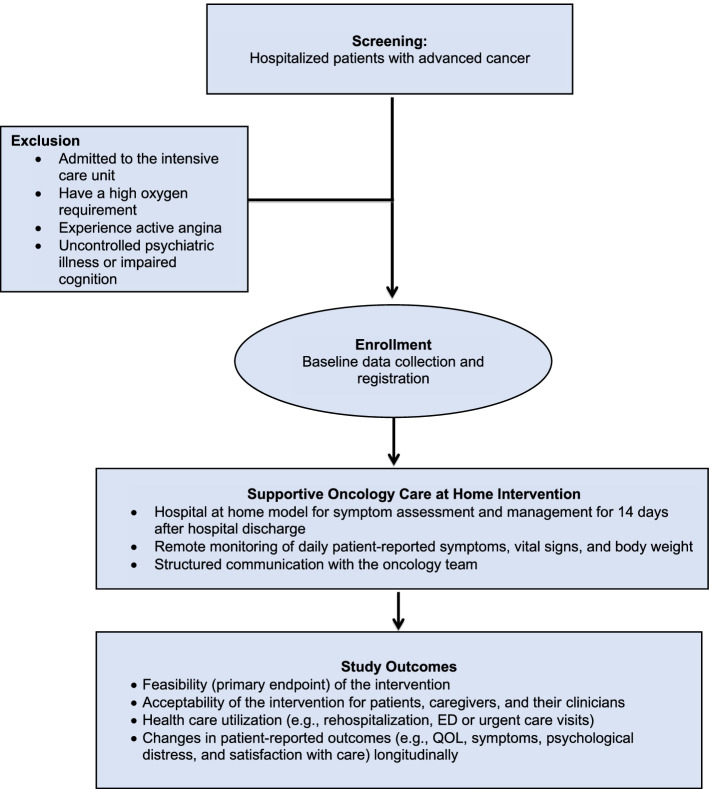
Supportive Oncology Care at Home for Hospitalized Patients with Cancer Consort Diagram

Hospitalized patients with advanced cancer represent a population at risk for high symptom burden and increased healthcare utilization. Elevated physical and psychological symptoms in hospitalized patients with advanced cancer are associated with increased healthcare utilization, including prolonged hospital stay and increased risk of readmissions [12, 14, 42]. We are conducting a single-arm pilot study (*N* = 30) to evaluate the feasibility and acceptability of the Supportive Oncology Care at Home intervention for recently hospitalized patients with advanced cancer and their caregivers (NCT04637035; DFCI Protocol Number:20–414). We will recruit hospitalized patients with advanced cancer from MGH. Figure 3 depicts the trial flow diagram. This study is approved by the DF/HCC Institutional Review Board.

#### Participant selection

To be eligible, patients must be (1) age 18 or older; (2) diagnosed with advanced cancer (defined as receiving treatment with palliative intent per chemotherapy order entry treatment intent designation and/or trial consent form or based on documentation in the oncology clinic notes for those not receiving chemotherapy); (3) admitted with an unplanned hospitalization at MGH; (4) not requiring intensive care unit-level care during their hospitalization; (5) verbally fluent in English; and (6) residing in the state of Massachusetts within approximately 50 miles of MGH. We will exclude patients who are (1) admitted to the intensive care unit; (2) have a high oxygen requirement (e.g., FiO2 > 0.40); (3) experience active angina or cardiac arrythmias during admission; (4) have a planned inpatient surgical or interventional procedure; (5) have uncontrolled psychiatric illness or impaired cognition interfering with their ability to understand study procedures and provide informed consent; (6) are deemed ineligible for home-based acute care based on the inpatient oncology clinician assessment; or (7) are planning to be discharged to hospice or to any location other than home.

Eligible patients may also identify caregivers for study participation. Caregivers must be (1) a relative or friend of eligible patient; (2) verbally fluent in English; and (3) age 18 or older. Patients without an available caregiver are still eligible to participate. The goal of this study entails assessing the acceptability of this care model, and thus we will also enroll the outpatient oncology physicians and advance practice clinicians who care for patients receiving Supportive Oncology Care at Home to obtain information regarding their perceptions of the intervention.

#### Enrollment

We will identify patients for study participation by screening the inpatient oncology census on a daily basis. We will recruit patients hospitalized at MGH within 72 h or three business days of admission. The study team will email the inpatient oncology team to request permission to approach potentially eligible patients for study participation. If the oncology clinician has no objections, the study team will approach the patient in-person or over the phone and obtain informed consent. Patients will then be asked to complete baseline assessment. We will ask patients interested in participating in the study to identify a caregiver upon whom they rely for help who might be willing to participate in the study. Caregivers will be eligible to enroll either at the same time as the patient, or within seven days of obtaining informed consent from the patient. Within one month of their patient completing the Supportive Oncology Care at Home intervention, study staff will meet with eligible clinicians, introduce them to the study, and obtain consent in a private setting.

#### Supportive oncology care at home intervention

Figure 1 depicts the Supportive Oncology Care at Home intervention as noted above. Participants will receive the intervention for a defined period of days to weeks (modified based on patient and clinician feedback) after their discharge from the hospital to reduce the risk of rehospitalization. Participants on study will continue to receive their standard oncology care and attend their regular clinic visits.

#### Study outcomes

The primary endpoint of this trial is feasibility. The proposed intervention will be deemed feasible if (1) at least 60% of eligible patients agree to participate in the study and (2) participants complete at least 60% of their daily assessments (i.e., patient-reported symptoms, vital signs, and body weight). Secondary endpoints include additional feasibility and acceptability metrics as well as participant-reported outcomes and healthcare utilization as depicted in Table 2. We will collect participant-reported outcomes at baseline and subsequent weeks post-enrollment.

**Table 2:** Supportive Oncology Care at Home for Hospitalized Patients with Cancer Study Outcomes

Study Outcomes
**Feasibility**
Proportion of patients who agree to participate in the study (goal at least 60%) **(primary)**
Proportion of daily assessments completed (goals at least 60%) **(primary)**
Number of home visits required, their average duration, the issues addressed at home, and the interventions delivered to patients at their home
Number of phone calls required per patient and their average duration
Number of emails from Medically Home to the primary oncology team
**Acceptability**
Acceptability ratings from patients, caregivers, and clinicians
Exit qualitative interviews with patients, caregivers, and their clinicians
**Health Care Utilization**
Index hospitalization length of stay
Proportion of days patients spent outside the hospital during the study period
Patients needing an urgent clinic visit (yes vs. no) and number of urgent clinic visits in the month post-enrollment
Patients needing an ED visit and number of ED visits in the month post-enrollment
Patients needing a hospitalization and number of hospitalizations in the month post-enrollment
Proportion of patients needing an urgent clinic visit, ED visit, or a hospitalization in the month post-enrollment
Hospitalization length of stay in the month post-enrollment
**Patient-reported outcomes**
Changes in symptom burden (ESAS) longitudinally throughout the study
Changes in QOL (FACT-G) longitudinally throughout the study
Changes in psychological distress (HADS) longitudinally throughout the study
Changes in care satisfaction (FAMCARE) longitudinally throughout the study
Changes in self-efficacy (PROMIS measures)
**Caregiver-reported outcomes**
Changes in caregiver QOL (CarGOQoL) longitudinally throughout the study
Changes in caregiving burden (CRA) longitudinally throughout the study

#### Sample size calculation

The primary endpoint of the proposed study is feasibility. We chose the sample size for this study based on the feasibility of completing the project during the appropriate timeframe. The proposed intervention will be deemed feasible if at least 60% of eligible patients will agree to participate and if participants complete at least 60% of daily patient-reported assessments during the study periods. These estimates are informed by our prior work in this population and consistent with other feasibility studies.

### Study #3: optimize end-of-life (EOL) care at home for patients with hematologic malignancies

#### Study design/objectives

There is a critical need to optimize EOL care in patients with hematologic malignancies, as data suggest that many patients with hematologic malignancies may not receive high quality EOL care [43–45]. Specifically, patients are often hospitalized during the last month of life and frequently die in the hospital [43–45]. Moreover, many patients die in the intensive care unit and receive chemotherapy during the last month of life [43–45]. Several barriers to optimizing EOL care in this population exist, including (1) the unique clinical circumstances, such as the high level of prognostic uncertainty and the intensity of therapy offered; (2) the rapid decline in patients’ health status at the EOL with unique complications such as bleeding/cytopenias and infections requiring intensive supportive care measures that are difficult to deliver at home; and (3) an insufficient exposure of hospice clinicians to patients with hematologic malignancies, which may limit their expertise in addressing patients’ EOL issues [43–48]. Thus, new models of EOL care delivery are needed to adequately meet the needs of this population and overcome barriers to optimizing their EOL care.

**Fig. 4:**
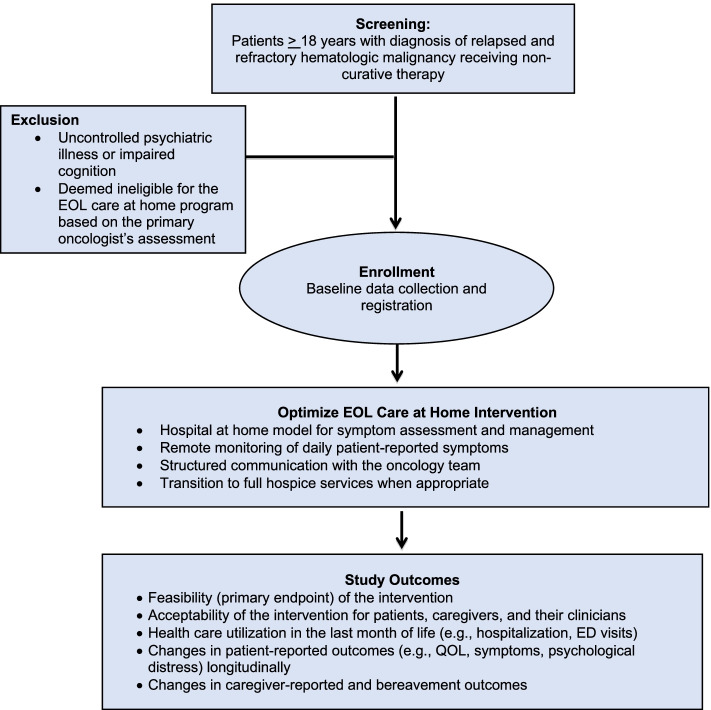
Optimize EOL Care for Patients with Hematologic Malignancies Consort Diagram

We are conducting a single-arm pilot study to evaluate the feasibility and acceptability of the Optimize EOL Care at Home intervention for patients with advanced hematologic malignancies and their caregivers (Fig. 4) (NCT04690205; DFCI Protocol Number:20–468). The specific aims of the trial are as follows: (1) to evaluate the feasibility of implementing Optimize EOL Care at Home for patients with advanced hematologic malignancies; (2) to determine the acceptability of Optimize EOL Care at Home for patients, caregivers, and clinicians; (3) to describe patient-reported outcomes and healthcare utilization at the EOL for patients with advanced hematologic malignancies receiving the intervention; and (4) to describe caregiver-reported outcomes and satisfaction with patients’ EOL care for caregivers of patients receiving the intervention. We are recruiting patients at MGH in Boston and MGH community affiliates (e.g., MGH Newton-Wellesley, MGH North Shore, MGH Waltham). This study is approved by the DF/HCC Institutional Review Board.

#### Participant selection

Eligibility criteria include (1) age 18 or older; (2) diagnosed with relapsed or refractory hematologic malignancy; (3) receiving treatment with non-curative intent or supportive care alone based on the intent of chemotherapy as reported in order entry or the Electronic Health Record; (4) deemed eligible to receive EOL care at home based on the primary oncologist’s assessment; (5) able to communicate and respond to questionnaires in English or with the assistance of an interpreter; and (6) residing in-state, within approximately 50 miles of MGH. Of note, patients requiring supportive transfusions in the oncology clinic will be eligible to participate. We will exclude patients with uncontrolled psychiatric illness or impaired cognition interfering with their ability to understand study procedures and provide informed consent, as determined by the primary oncologist.

Eligible patients may also identify caregivers for study participation. Caregivers must be (1) a relative or friend of eligible patient; (2) verbally fluent in English; (3) age 18 or older; and (4) involved in the care of patients near the EOL. Patients without an available caregiver are still eligible to participate. Thus, up to 30 caregivers can participate in the study. Our goal is to assess the acceptability of this care model, and we will therefore enroll the outpatient oncology physicians and advance practice clinicians who care for patients receiving the Optimize EOL Care at Home intervention to obtain their feedback on the intervention.

#### Enrollment

Patient recruitment will rely primarily on referral from oncology clinicians caring for patients with hematologic malignancies. Study staff will attend the weekly team meetings for the lymphoma, leukemia, myeloma, and stem cell transplantation and cellular therapy teams to ensure that eligible patients are identified in a timely fashion. Study staff will inquire during the team meetings regarding any potential patients who might be eligible for the study. The oncology team will then introduce the study to the patient. Subsequently, the study team will approach the patient in-person or over the phone and obtain informed consent. We will then ask patients to complete baseline assessments. We will ask patients interested in participating in the study to identify a caregiver upon whom they rely for help who might be willing to participate in the study. Caregivers will be eligible to enroll either at the same time as the patient, or within seven days of patient consent.

Within one month of study completion, study staff will meet with eligible clinicians, introduce them to the study, and obtain consent in a private setting. We anticipate approximately 15 clinicians will participate in the study.

#### Optimize eol care at home intervention

The Optimize EOL Care at Home intervention components are similar to those described above in Fig. 1. Participants will receive the intervention from the time of enrollment until death. This population experiences a substantial care burden, and thus we will only obtain daily patient-reported symptom assessments. The vital signs and body weight assessments will be utilized as needed based on the Medically Home clinicians’ assessments. Patients are permitted to attend their oncology visits, receive supportive transfusions, or palliative chemotherapy without any restrictions on the care provided by the oncology team. For patients receiving the intervention who transition to receive full hospice services, the Medically Home team will provide hospice services as part of their care in collaboration a hospice program.

#### Study outcomes

The primary endpoint of this trial is feasibility. The proposed intervention will be deemed feasible if (1) at least 60% of eligible patients agree to participate in the study and (2) at least 80% of enrolled participants complete a minimum of 60% of their daily patient-reported symptoms. Secondary endpoints include additional feasibility and acceptability metrics as well as participant-reported outcomes and healthcare utilization as depicted in Table 3. Participant-reported outcomes will be collected longitudinally every three weeks throughout the study period.

**Table 3:** Optimize EOL Care for Patients with Hematologic Malignancies Study Outcomes

Study Outcomes
**Feasibility**
Proportion of patients who agree to participate in the study (goal at least 60%) **(primary)**
Proportion of participants who complete a minimum of 60% of their daily patient-reported assessments (goal at least 80%) **(primary)**
Number of home visits required, their average duration, the issues addressed at home, and the interventions delivered to patients at their home
Number of phone calls required per patient and their average duration
Number of emails from Medically Home to the primary oncology team
**Acceptability**
Acceptability ratings from patients, caregivers, and clinicians
Exit qualitative interviews with patients, caregivers, and their clinicians
**Health Care Utilization**
Proportion of patients requiring a hospitalization in the lats week and month of life
Number of hospitalizations in the last month of life
Proportion of patients needing an urgent clinic visit in the last month of life
Number of urgent clinic visits in the last month of life
Proportion of patients needing an ED visit in the last month of life
Number of ED visits in the last month of life
Death location
Proportion of days patients spend outside of the hospital or clinic in the last month of life
Proportion of patients needing an urgent clinic visit, ED visit, or a hospitalization throughout the study period
**Patient-reported outcomes**
Changes in symptom burden (ESAS) longitudinally throughout the study
Changes in QOL (FACT-G) longitudinally throughout the study
Changes in psychological distress (HADS) longitudinally throughout the study
**Caregiver-reported outcomes**
Changes in caregiver QOL (CarGOQoL) longitudinally throughout the study
Changes in caregiving burden (CRA) longitudinally throughout the study
Changes in psychological distress (HADS) longitudinally throughout the study
Caregiver satisfaction with patient’s EO care (FAMCARE)

#### Sample size calculation

The primary endpoint of the proposed study is feasibility. We chose the sample size for this study based on the feasibility of completing the project during the appropriate timeframe. The proposed intervention will be deemed feasible if at least 60% of eligible patients agree to participate and at least 80% of enrolled patients complete a minimum of 60% of their daily patient-reported symptom assessments during the study period.

## Discussion

The goal of the Supportive Oncology Care at Home research platform is to transform the paradigm of care for patients with cancer by providing them with the necessary support at home to improve their health outcomes and care delivery. The clinical trials outlined in this manuscript represent an innovative strategy to evaluate the feasibility, acceptability, and efficacy of these longitudinal care models throughout the continuum of illness for patients with cancer. Although these trials represent our initial efforts to determine the role of Supportive Oncology Care at Home, continued scalability and reproducibility of this care model will require diligent efforts at maintaining consistency of care quality across remote settings, which these early studies will help to inform. For example, we have developed a treatment manual that will help to foster reproducibility and assist with scalability. Additionally, we will incorporate feedback from patients, caregivers, and clinicians to help understand the perceptions of each of these key stakeholders, which will guide necessary modifications to the intervention, including how best to integrate caregivers into future iterations of this work. Furthermore, future work will focus on testing other home-based interventions to provide high-quality oncology care for patients with cancer, including transfusion support, chemotherapy administration, as well as maximal supportive care. Importantly, hospital at home care models have the capability of enhancing access to oncology care in underserved and rural communities and provide scalable solutions to optimize the quality of cancer care across the globe. Effectively broadening the geographic reach of Supportive Oncology Care at Home will require efforts to first understand the unique needs of patients across differing health systems, while also working to expand the workforce and remote monitoring capabilities to meet the distinct needs of individuals in more remote regions. Collectively, this work will expand hospital at home care from an acute care strategy replacing hospitalizations to a longitudinal care delivery model with tremendous potential to revolutionize the paradigm of care for patients with cancer.

## Data Availability

Not applicable.

## Abbreviations

ED: Emergency Department
QOL: Quality of life
ESAS: Edmonton Symptom Assessment System
MGH: Massachusetts General Hospital
DF/HCC: Dana-Farber/Harvard Cancer Center
EOL: End-of-Life
